# Clear cell carcinoid tumor of the distal common bile duct

**DOI:** 10.1186/1477-7819-5-6

**Published:** 2007-01-17

**Authors:** Takeshi Todoroki, Takaaki Sano, Shuji Yamada, Nobutsune Hirahara, Naotaka Toda, Katsuhiko Tsukada, Ryuji Motojima, Teiji Motojima

**Affiliations:** 1 Department of surgery Motojima General Hospital, Ota, 373-0033, Japan; 2 Department of Tumor pathology, Graduate School of Medicine, Gunma University, Maebashi, 371-8511, Japan; 3 Department of gastroenterology, Motojima General Hospital, Ota, 373-0033, Japan

## Abstract

**Background:**

Carcinoid tumors rarely arise in the extrahepatic bile duct and can be difficult to distinguish from carcinoma. There are no reports of clear cell carcinoid (CCC) tumors in the distal bile duct (DBD) to the best of our knowledge. Herein, we report a CCC tumor in the DBD and review the literature concerning extrahepatic bile duct carcinoid tumors.

**Case presentation:**

A 73-old man presented with fever and occult obstructive jaundice. Ultrasonography, computed tomography (CT) and magnetic resonance cholangiopancreaticography (MRCP) demonstrated a nodular tumor projection in the DBD without regional lymph node swelling. Under suspicion of carcinoma, we resected the head of the pancreas along with 2^nd ^portion duodenectomy and a lymph node dissection. The surgical specimen showed a golden yellow polypoid tumor in the DBD (0.8 × 0.6 × 0.5 cm in size). The lesion was composed of clear polygonal cells arranged in nests and a trabecular pattern. The tumor invaded through the wall into the fibromuscular layer. Immunohistochemical stains showed that neoplastic cells were positive for neuron-specific enolase (NSE), chromogranin A, synaptophysin, and pancreatic polypeptide and negative for inhibin, keratin, CD56, serotonin, gastrin and somatostatin. The postoperative course was uneventful and he is living well without relapse 12 months after surgery.

**Conclusion:**

Given the preoperative difficulty in differentiating carcinoid from carcinoma, the pancreaticoduodenectomy is an appropriate treatment choice for carcinoid tumors located within the intra-pancreatic bile duct.

## Background

Extrahepatic bile duct carcinoid tumors are extraordinarily rare lesions and account for only 0.32% of all carcinoids of the gastrointestinal tract [1]. Since 1961 when Pilz described the first carcinoid tumor arising from the common bile duct, only 51 cases have been described, excluding the ampulla of Vater, cystic duct, gallbladder and intrahepatic bile ducts. In particular, the morphologically distinct primary clear cell carcinoid tumor of the extrahepatic bile duct is even rarer; there is no previous report to the best of our knowledge.

We report herein a clear cell carcinoid tumor of arising from the distal (intra-pancreatic portion) common bile duct as the first report of a clear cell carcinoid tumor of the extrahepatic bile duct. Because of the rarity of carcinoid tumors in the bile duct as well as the rarity of clear cell carcinoid tumors, it is difficult to adequately assess their malignant potential, their responsiveness to particular treatments and their overall prognosis. Improved understanding can only be obtained by analyzing the collected data from reported cases; accordingly, we reviewed the literature concerning carcinoid tumors originating from the extrahepatic bile ducts.

## Case presentation

**Figure 1:**
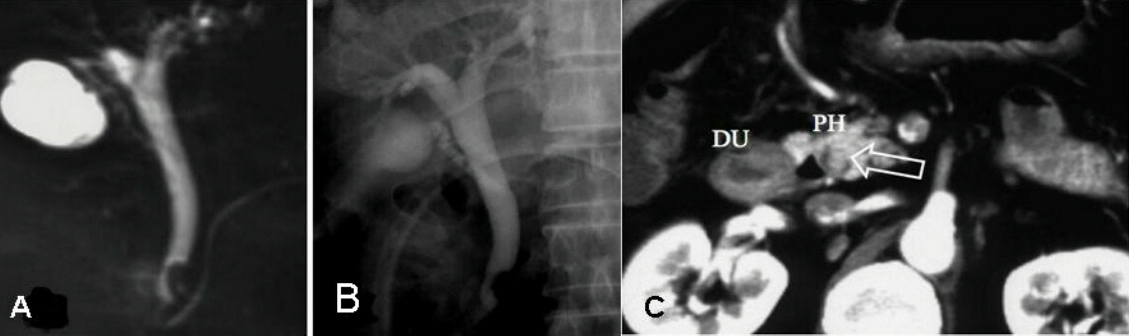
**Cholangiographies**: A and B). Endoscopic retrograde cholangiopancreatography (ERCP) and C). Magnetic resonance cholangiopancreatography (MRCP) showing a nodular mass protruding into the bile duct lumen from the left wall at the distal end of the common bile duct.

A 73-old man was visited our hospital with high fever (39°C), epigastric pain and reduced consciousness. He had suffered from poorly controlled diabetes mellitus, hypertension and multiple brain infarctions during the previous 3 years. On physical examination, no abdominal mass was palpable. Laboratory tests revealed signs of inflammation, including a WBC count of 34,400/mm3 and a CRP 10.4 of mg/dl, and abnormal liver function results with signs of biliary obstruction (AST/ALT 418/479 IU/L, ALP 591IU/L, γGTP 1041 IU/L, and TBL/DBL 4.2/2.9 mg/dl). The tumor markers were within the normal range: CEA 4.0 ng/ml and CA19-9 128 U/ml (slight elevation). The patient's blood sugar was elevated at 311 mg/dl with an elevated HbA1C of 7.9%, but no ketone bodies were detected in the urine. Contrast-enhanced abdominal CT revealed a slight dilatation of the intra/extra-hepatic bile ducts, a slightly enhanced protruding nodular lesion in the intra-pancreatic common bile duct and no lymph node swelling neither around the head of pancreas nor within the hepato-duodenal ligament. Ultrasonography demonstrated a slightly thickened gallbladder wall without calculi. Endoscopic retrograde cholangiopancreatography (ERCP) and MRCP showed a nodular mass with an irregular surface protruding into the bile duct lumen from the left wall at the distal end of the common bile duct (Fig. 1A&1B ). Abdominal arteriography and portography demonstrated no abnormalities and we carried out surgical resection with suspicion of carcinoma less than stage II of the intra-pancreatic bile duct.

During laparotomy, a polypoid lesion was palpated in the common bile duct at the head of the pancreas. However, whether tumor was invading the pancreas or not was unclear and regional nodes were soft and slightly swollen. Given the suspicion of distal bile duct carcinoma without node metastasis (less than stage II); the patient underwent a modified pancreaticoduodenectomy. Taking into account possible complications, we tried to preserve not only the pylorus, but also the first and third portions of the duodenum and performed a regional lymphadenectomy.

**Figure 2:**
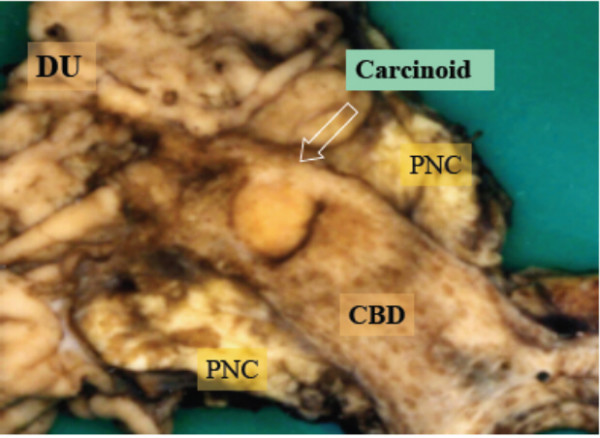
The surgical specimen showed a golden yellowish polypoid mass (arrow), measuring 1.0 × 1.2 × 0.7 cm in size, projecting from the surface of the intra-pancreatic portion of the bile duct. DU: duodenum, PNC: pancreas head, CBD: common bile duct, GB: gallbladder

**Figure 3:**
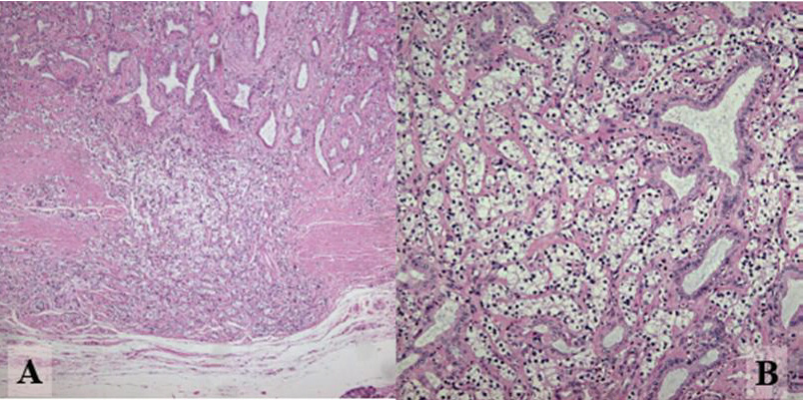
**Photomicrographs stained by Hematoxylin and Eosin**. A: Tumor cells penetrating the fibromuscular layer of the bile duct, but not reaching to the surrounding pancreatic parenchyma. (Original magnification ×200). B: Tumor consisting of fairly uniform polygonal cells in size, with round nuclei and clear and abundant cytoplasm. Neoplastic cells are arranged in combination patterns with solid nests and trabecular growth. Preexisting non-neoplastic gland is entrapped in the lesion. (Original magnification ×400)

**Figure 4:**
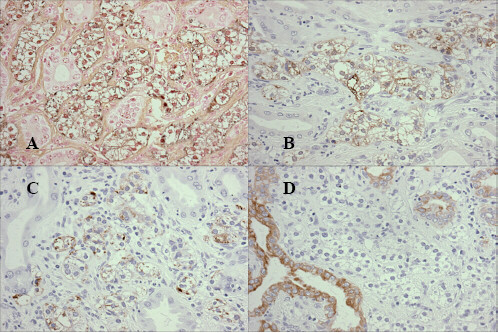
Tumor cells were stained by Grimelius silver (A), and positive for immunohistochemical staining of Chromogranin-A (B) and for Synaptophysin (C). Clear cells were completely negative for Keratin, but positive in the intercalated non-neoplastic glands (D).

Macroscopically, the surgical specimen showed a golden yellowish polypoid mass, measuring 1.0 × 1.2 × 0.7 cm in size that was projecting from the surface of the intra-pancreatic portion of the bile duct. The superficial yellow discoloration extended to the proximal epithelium from the tumor base. The thickening of the bile duct wall at the tumor bed suggested longitudinal and vertical tumor invasion (Figure 2). Histologically, the tumor consisted of fairly uniformly sized polygonal cells, with small round nuclei, inconspicuous nucleoli, and clear abundant cytoplasm. The neoplastic cells were arranged in combination patterns with tubular or trabecular anastomozing structures and solid nests. The tumor cells penetrated the fibromuscular layer of the bile duct, but did not reach the surrounding pancreatic parenchyma (Figure 3). Mitotic figures were not visible. There were no necrotic areas and no vascular invasion, but there was limited perineural invasion and were questionable areas of lymphovascular invasion. The regional lymph nodes and resection margins were negative for tumor cell invasion. Tumor cells showed positive staining for Grimelius silver. Immunohistochemical stains were positive for various neuroendocrine markers as chromogranin A, synaptophysin, neuron-specific-enolase, and pancreatic polypeptide (Fig. 4). However, inhibin, keratin, CD56, somatostatin, serotonin, and gastrin were negatively stained. Based on these findings, we diagnosed the patient with a clear cell carcinoid tumor of the distal (intra-pancreatic) common bile duct. The postoperative course was uneventful and the patient is well with no evidence of relapse 12 months after surgery.

## Discussion

Recently, the World Health Organization (WHO) developed a new classification system that gives a better description of the characteristics and biological behavior of the carcinoid tumors. These tumors can be malignant and the histopathological differentiation of carcinoid tumors ranges from well differentiated (traditionally known as carcinoid tumors) to poorly differentiated malignancies with neuroendocrine features, typified by small cell carcinoma. Carcinoid tumors derived from neuroendocrine cell compartments and their frequency of occurrence correlate with the site-density of neuroendocrine cells. Most (over 60%) of carcinoid tumors arise from the gastrointestinal system, followed by the bronchopulmonary system (25%). Carcinoid tumors of the extrahepatic bile duct, excluding Vater, intrahepatic bile duct, gallbladder and cystic duct, are extremely rare. The incidence in all gastrointestinal carcinoid tumors is 0.32%, according to the recent analysis of 13,715 carcinoid tumors [1]. On the other hand, more than 80% of the extrahepatic bile duct tumors are adenocarcinomas [2,3]. Carcinoid tumors are slow-growing malignancies, as was first emphasized by the use of the term "carcinoid" in 1907, referring to the benign nature of the tumor [4]. Pilz reported the first case of a carcinoid tumor of the common bile duct in 1961 [5]; thereafter, 51 cases of extrahepatic bile duct carcinoid tumors, excluding composite tumors with carcinoma, have been reported in the literature (See Additional file 1). Reports by Davies [6] and Godwin [7] were excluded because of the lack of detail. Exploring the original site of the tumor in detail, only 11 cases were found to have arisen from the intra-pancreatic (distal) portion (DBD), 14 cases arose in the middle portion (MBD) and the remaining 26 cases arose in the proximal bile ducts (PBD), including the right, left and common hepatic ducts (see additional file 1).

Only two clear cell carcinoid (CCC) tumors of the gallbladder have been described as originating from the biliary tracts; however, there have been no case reports of CCCs arising from the extrahepatic bile ducts. One case has been interpreted to be a CCC tumor of the gallbladder and appeared to be a distinctive manifestation of von Hippel-Lindau (VHL) disease with diffuse expression of inhibin in the tumor [8]. Those authors hypothesized that inhibin may be a product of CCC tumors associated with VHL disease and a useful marker in distinguishing these from metastatic renal cell carcinomas. On the other hand, in another case the patient's tumor was not associated with VHL disease and the CCC tumor had negative expression of inhibin [9]. In our case, the CCC tumor was also negative for inhibin without any correlation to VHL disease. Although further investigations of inhibin expression in CCC tumors of the biliary tract with and without VHL disease are required, Sinkre's hypothesis is an interesting in suggesting a biomarker to detect concomitant VHL disease.

Carcinoid tumors are composed of multipotential cells with the ability to secrete numerous hormonal substances and vasoactive peptides. These substances cause the clinical features that constitute carcinoid syndrome; however, within the extrahepatic bile duct, only one carcinoid tumor has been associated with the syndrome [10]. The most popular symptom for extrahepatic bile duct carcinoids was obstructive jaundice, accounting for 75 percent of the reported cases. From the viewpoint of treatment, the prime concern is the location of the tumor and the malignant potential of invasion and metastasis rather than the presence of the carcinoid syndrome. When the distal (intra-pancreatic) bile duct is affected, surgery should consist of a head of pancreas resection with a whole duodenectomy (pancreaticoduodenectomy) or a resection of the second portion of the duodenum, as well as pyloric preservation to achieve adequate radicality. In our case, a pancreatic head resection with a 2^nd ^portion duodenectomy was successfully performed with an *en bloc *resection of the tumor and surrounding structures including regional lymph nodes. In the event of middle to proximal bile duct involvement, an *en bloc *excision of the bile ducts from the main hepatic ducts down to the upper margin of the pancreas and adjoining lymph nodes (with construction of a Roux-en-Y hepaticojejunostomy or hepaticoduodenostomy) will be appropriate to preserve the patient's prospects for a cure.

Accurate pre-and intra-operative histological diagnosis may be problematic because the discrimination between carcinoid tumors and carcinoma is difficult at times, particularly with frozen section specimens. In some cases, frozen section diagnosis is inconclusive and resection must be undertaken with an indeterminate diagnosis. In our case, cytological examination failed to demonstrate carcinoid cells. However, imaging diagnostic findings obtained from CT, MRCP and ERCP and abdominal angiography were actually regarded as sufficient evidence to precede radical surgery since those images demonstrated a protruding nodular tumor in the distal bile duct and no evidence of metastasis in the regional nodes, distant metastasis and vascular involvement.

Carcinoid tumors are slow-growing malignancies; however, prognosis after treatment depends on the evidence of degree of malignancy. Mitotic counts and the degree of anaplasia cannot reliably determine malignancy; rather, it is microscopic local invasion or gross distant spreads that differentiate the benign from the malignant forms of this disease. Metastases to distant organs (chiefly the liver), regional lymph nodes and direct invasion into the perineural lymph space, microscopic vessels and adjacent structures were found in 21 of 49 reported cases (including our case and excluding three cases which lacked adequate information). Out of those 21 patients with extended tumors, only two deaths)[11,12] were directly attributable to progression of the carcinoid tumors after radical surgery, although one patient died from its tumor one day after celiotomy for carcinoidomatosis [13]. On the other hand, one patient with a liver metastasis and 15 patients with loco-regional extension are living well 18 months later [14], and from 6 months to 20 years [15] after radical resection surgery, respectively (see Additional file 1). The details of earlier reported case are illustrated in additional file 1[16-52]. However, because of the rarity of carcinoid tumors of the bile ducts, it is difficult to accurately and adequately assess the natural history of this disease, the malignant potential of the tumors, their responsiveness to particular treatments, and the overall prognosis after different types of therapy. This can only be achieved with continued reporting of cases in detail.

## Conclusion

Definite diagnosis is difficult preoperatively but may be improved with the assessment of neuroendocrine markers in suspected cases. Disease-free survival is prolonged following complete and safe resection of the tumor. Because carcinoid tumors are slow growing and less aggressive than adenocarcinoma, even if these patients have metastases, they should be treated aggressively. Continued reporting of single cases and long-term follow-up should be encouraged with reporting of the results of treatment.

## Competing interests

The author(s) declare that there are no competing interests

## Authors' contributions

**TS **carried out the pathologic studies, participated in the sequence alignment and drafted the manuscript. **SY **participated in surgical operation as one of assistants for **TT **and took charge of post-operative managements together with **KT. NH, NT **and **RM **carried out the preoperative diagnostic work-up and corporate design of the study in cooperation with **TM**.

All authors read and approved the final manuscript.

## Supplementary Material

Additional file 1 showing case review of carcinoid tumors of the extrahepatic bile ducts. Click here for file
