# Supplementary material for: Clear cell carcinoid tumor of the distal common bile duct
Source: World J Surg Oncol. 2007 Jan 17;5:6. doi: 10.1186/1477-7819-5-6 (PMC1785380; doi:10.1186/1477-7819-5-6)
Supplement: Additional file 1 — showing case review of carcinoid tumors of the extrahepatic bile ducts. [file 1477-7819-5-6-S1.doc]

**Additional file 1. case review of carcinoid tumors of** the extrahepatic bile ducts

| **No** | **Author**  **(Year)** | **Gender**  **Age** | **Symptoms** | **Site** | **Positive**  **Staining** | **Treat**  **ments** | **Outcome** |
| --- | --- | --- | --- | --- | --- | --- | --- |
| 1 | Pilz[5]  (1961) | F  55 | Abd. Pain  Jaundice | MBD | Argentaffin | TR, T-tube | AWOD,  1.5-yrs. |
| 2 | Little[16]  (1968) | F  41 | Abd. Pain  Jaundice | PBD | Argentaffin  Diazonium | Biopsy/  bypass | DOC |
| 3 | Bergdahl[17] (1976) | F  79 | Fever | DBD | N/A | No | DOD, 1-day |
| 4 | Gerlock [18] (1979) | M  32 | Painless jaundice | MBD | N/A | BDR | N/A |
| 5 | Nakamura [19] (1981) | F  58 | Jaundice | MBD | Argyrophilic granule | BDR | DOC, 6-days |
| 6 | Vitaux [20]  (1981) | M  30 | Jaundice | DBD | Argentaffin | PD | AWOD,  48-Mon. |
| 7 | Abe [11]  (1983) | M  64 | Abd. Pain | DBD | Argyrophilia | PD | DOD, LVM. 10-Mon. |
| 8 | Alexander[21] (1986) | F  64 | GI bleeding | PBD  (Hilar) | N/A | BDR | AWOD,  8-Mon. |
| 9 | Jutte [22]  (1987) | M  62 | LBP | PBD  (Hilar) | Argyrophilia | BDR | AWOD,  2.5-Mon. |
| 10 | Bickerstaff [23](1987) | F  57 | Jaundice  Lethargy | MBD | Argyrophilia | PD | AWOD,  6^-^Mon. |
| 11 | Gastinger [24](1987) | F  65 | Abd. Pain  Jaundice | PBD  (Hilar) | Argyrophilia | TR | N/A |
| 12 | Reinhardt [25] (1988) | F  71 | Jaundice  Fever | DBD | Chromogranin, NSE | PD | AWOD,  2^-^Mon. |
| 13 | Fujita [26]  (1989) | F  55 | Abd. Pain | PBD  (Hilar) | Argyrophilia | TR, T-tube | AWOD,  6^-^Mon. |
| 14 | Bumin [27]  (1990) | F  39 | Abd. Pain Jaundice | PBD  (Hilar) | N/A | TR, T-tube | AWOD,  40^-^days |
| 15 | Brown[28]  (1990) | F  35 | Jaundice | PBD  (Hilar) | Chromogranin, Serotonin, NSE, EMA, Keratin | BDR | AWOD,  7-days |
| 16 | Fellows[29]  (1990) | M  30 | Jaundice | MBD | S-100, PGP 9.5, CSTK, Gastrin, Somatostatin | BDR | AWOD,  6^-^wks. |
| 17 | Besznyak [30] (1990) | 13  F | Jaundice | PBD  (Hilar) | N/A | RHLB | AWOD,  12-yrs. |
| 18 | Barron-  Rodriguez [13](1991) | M  36 | Jaundice  Vomiting  Fever | PBD  (Hilar) | Argyrophilia  Chromogranin Synaptophysin, NSE, Keratin, leu7 | Biopsy | DOC,  4-days |
| 19 | Angeles-  Angeles[31]  (1991) | F  39 | Jaundice  Abd. Pain Fever | MBD | Argyrophilia  Chromogranin Serotonin  Somatostatin | BDR | AWOD,  11-Mon. |
| 20 | Newman[32](1992) | F  15 | N/A | DBD | Serotonin  Glucagon, PP | PpPD | AWOD,  4-yrs. |
| 21 | Dixon[33]  (1992) | F  60 | Abd. Pain | MBD | N/A | TR, T-tube | AWOD,  17-Mon. |
| 22 | Rugge[34]  (1992) | F  64 | Jaundice  Abd. Pain | MBD | EMA, Keratin  Chromogranin | BDR | AWOD,  12-Mon. |
| 23 | Mandujano-Vera[10]  (1995) | F  53 | Jaundice  Abd. Pain | DBD | Gastrin  Chromogranin  Synaptophysin, NSE, PP, Serotonin | PD | AWOD,  6-yrs. |
| 24 | Sankary[35]  (1995) | F  47 | Jaundice | PBD  (Hilar) |  | Trisegmentectomy | AWOD,  4-yrs. |
| 25 | Belli[36]  (1996) | M  78 | Jaundice | PBD  (Hilar) | Chromogranin | BDR | AWOD,  15-Mon. |
| 26 | Kopelman [14](1996) | M  44 | Jaundice | DBD | Chromogranin | PpPD | AWOD,  18-Mon. |
| 27 | Hao[37]  (1996) | M  42 | No | MBD | Chromogranin  Gastrin, Serotonin | OLTP | AWOD,  5-Mon. |
| 28 | Nahas [38]  (1998) | M  61 | Jaundice  Abd. Pain | PBD  (Hilar) | S-100 | BDR | AWOD,  6-Mon. |
| 29 | Bembenek [39] (1998) | F  12 | Jaundice  Abd. Pain | PBD  (Hilar) | Chromogranin  Gastrin, NSE, Serotonin | BDR | AWOD,  9-Mon. |
| 30 | Perakath [40] (1999) | F  36 | Jaundice  Abd. Pain | PBD  (Hilar) | NSE | BDR | AWOD,  6-Mon. |
| 31 | Ross [41]  (1999) | F  65 | Jaundice | DBD | Chromogranin  NSE, | PD | AWOD,  17-Mon. |
| 32 | Chamberlain [42](1999) | F  37 | Itching | PBD  (Hilar) | Chromogranin  Synaptophysin  PP | BDR | AWOD,  18-Mon. |
| 33 |  | F  67 | Itching | PBD  (Hilar) | N/A | BDR | AWOD,  15-Mon. |
| 34 | Maitra [43]  (2000) | N/A  42 | Jaundice  Pruitis | MBD | Chromogranin  Somatostatin | BDR | AWOD,  11-yrs. |
| 35 |  | N/A  61 | Jaundice  Pruitis | PBD  (Hilar) | Chromogranin  Serotonin | BDR | AWOD,  4-yrs. |
| 36 |  | N/A | Jaundice  Pain | MBD | Chromogranin  Gastrin | BDR | AWOD,  10-yrs. |
| 37 |  | N/A  37 | Pruitis | PBD  (Hilar) | Chromogranin  Synaptophysin  PP | BDR | AWOD,  2-yrs. |
| 38 |  | N/A  67 | No | PBD  (Hilar) | Chromogranin | BDR | AWOD,  2-yrs. |
| 39 | Chan [44]  (2000) | M  14 | Jaundice | PBD  (Hilar) | Chromogranin  Synaptophysin  Serotonin, VIP  Gastrin | BDR | N/A |
| 40 | Jutri [45]  (2000) | M  43 | GUAP,  Jaundice | DBD | Chromogranin  Gastrin | PD | AWOD,  3.5-yrs. |
| 41 | Volpe [46]  (2003) | M  19 | Jaundice  Pain | PBD  (Hilar) | Chromogranin | BDR | AWOD,  1-Yrs. |
| 42 | Podnos [47]  (2003) | F  65 | Cholecystitis | MBD | Chromogranin  NSE | BDR | AWOD,  37-Mon. |
| 43 |  | M  27 | Jaundice  SCCGT | MBD | NA | OLTP | AWOD, 7.5-yrs. |
| 44 | Pawlik [48]  (2003) | M  59 | Jaundice | PBD  (LHBD) | N/A | BDR | AWOD,  6-Mon. |
| 45 | Rassi [15]  (2004) | F  49 | Jaundice | PBD  (Hilar) | Argentaffin G,  Chromogranin | LHLB | AWOD,  20-yrs. |
| 46 |  | M  79 | Jaundice | DBD | Argyrophilic  Chromogranin | PpPD | DOD, LVM, 2.8-yrs. |
| 47 | Menezes [49] (2004) | M  30 | Jaundice | PBD | NSE | BDR | AWOD,  18-Mon. |
| 48 | Ligato [50]  (2005) | F  33 | Irritable bowel | PBD  (Hilar) | Gastrin | BDR | AWOD,  10-Mon. |
| 49 | Nesi [51]  (2006) | M  30 | Jaundice  Diarrhea | DBD | Chromogranin  Synaptophysin  Cytokeratin, NSE, Serotonin | PpPD | AWOD,  7-yrs. |
| 50 | Kim [52]  (2006) | F  67 | Jaundice | MBD | Chromogranin  Synaptophysin  CD56 | PpPD | AWOD,  10-Mo. |
| 51 | Caglikulekci[12] (2006) | F  40 | Jaundice | PBD  (Hilar) | Chromogranin | BDR  Chemo. (Cis) | DOD,  14-Mon. with PLM, LVM, PER |
| 52 | Present study | M  73 | Fever  Abd. Pain | DBD | Chromogranin  Synaptophysin  NSE | PD | AWOD,  12-Mon. |

Abd.: abdominal, MBD; middle bile duct, TR.; tumor resection, AWOD; alive without disease, DOC; dead of other cause, DOD; dead of disease, yr.; year, , PBD; proximal bile duct, DBD; distal (intra-panceratic) bile duct, N/A; not available, BDR; bile duct resection, PD; pancreaticoduodenectomy, Mo.: month, LVM; liver metastases, GI; gastrointestinal, LBP; lower back pain, NSE; neuron-specific enolase, EMA; epithelial membrane antigen, CSTK; cholecystokinin, RHBD; right hepatic bile duct, RHLB; right hepatic lobectomy, PP; pancreatic peptide, PpPD; pylorus preserving PD, OLTP; orthotopic liver transplantation**,** VIP; vasoactive intestinal peptide; LHLB: left hepatic lobectomy, PLM; pulmonary metastasis, PER; peritoneal metastasis, Cis; cis-platinum
